# Whole genome sequencing unveils genetic heterogeneity in optic nerve hypoplasia

**DOI:** 10.1371/journal.pone.0228622

**Published:** 2020-02-10

**Authors:** Sara Dahl, Maria Pettersson, Jesper Eisfeldt, Anna Katharina Schröder, Ronny Wickström, Kristina Teär Fahnehjelm, Britt-Marie Anderlid, Anna Lindstrand

**Affiliations:** 1 Department of Women’s and Children’s Health, Neuropediatric Unit, Karolinska Institutet, Stockholm, Sweden; 2 Department of Molecular Medicine and Surgery, Center for Molecular Medicine, Karolinska Institutet, Stockholm, Sweden; 3 Science for Life Laboratory, Karolinska Institutet Science Park, Stockholm, Sweden; 4 Department of Paediatric Ophthalmology, St. Erik Eye Hospital, Stockholm, Sweden; 5 Department of Clinical Neuroscience, Karolinska Institutet, Stockholm, Sweden; 6 Department of Clinical Genetics, Karolinska University Hospital, Stockholm, Sweden; German Cancer Research Center (DKFZ), GERMANY

## Abstract

Optic nerve hypoplasia (ONH) is a congenital malformation with a reduced number of retinal ganglion cell axons in a thin optic nerve. It is a common cause of visual impairment in children and ONH is associated with neurodevelopmental disorders, pituitary hormone deficiencies, and brain malformations. In most cases, the aetiology is unknown, but both environmental factors and genetic causes have been described. This study aimed to identify genetic variants underlying ONH in a well-characterised cohort of individuals with ONH. We performed array comparative genomic hybridization and whole genome sequencing in 29 individuals with ONH. Rare variants were verified by Sanger sequencing and inheritance was assessed in parental samples. We identified 11 rare single nucleotide variants (SNVs) in ten individuals, including a homozygous variant in *KIF7* (previously associated with Joubert syndrome), a heterozygous *de novo* variant in *COL4A1* (previously described in an individual with porencephaly), and a homozygous variant in *COL4A2*. In addition, one individual harboured a heterozygous variant in *OPA1* and a heterozygous variant in *COL4A1*, both were inherited and assessed as variants of unknown clinical significance. Finally, a heterozygous deletion of 341 kb involving exons 7–18 of *SOX5* (associated with Lamb-Schaffer syndrome) was identified in one individual. The overall diagnostic yield of pathogenic or likely pathogenic variants in individuals with ONH using whole genome sequencing was 4/29 (14%). Our results show that there is a genetic heterogeneity in ONH and indicate that genetic causes of ONH are not rare. We conclude that genetic testing is valuable in a substantial proportion of the individuals with ONH, especially in cases with non-isolated ONH.

## Introduction

Optic nerve hypoplasia (ONH) (MIM 165550) is a congenital ocular malformation with a thin optic nerve due to a reduced number of retinal ganglion cell axons. It is the most common optic nerve malformation [1,2] and one of the most common causes of visual impairment and blindness in children in developed countries [3]. It can be unilateral or bilateral and results in a visual outcome ranging from near normal to blindness. In children with severe bilateral ONH, the most common presenting symptoms are lack of fixation, poor visual behaviour, roving eye movements, nystagmus, and strabismus [1]. ONH is diagnosed by examining the optic disc by ophthalmoscopy or in fundus photographs. The prevalence has been reported to be 10.9/100 000 in Northwest England [4] and 17.3/100 000 in Stockholm, Sweden [1]. Individuals with ONH often have coexisting pituitary hormone deficiencies, intellectual disability, autism, and other brain malformations [3,5,6].

The aetiology of ONH is unknown in the majority of cases. Predominant maternal factors are young maternal age and primiparity, and environmental factors have been suspected [7]. Congenital infections have been associated with ONH, as has prenatal ethanol exposure as a part of the fetal alcohol syndrome [3]. Furthermore, destructive brain lesions have been suggested to cause ONH and associated brain malformations [8]. Although most cases of ONH are sporadic, there are also familial cases suggesting a genetic aetiology [9,10]. *HESX1* was the first gene to be associated with ONH [10], but only few cases have been reported [11,12]. Due to the increasing rate of genetic testing of affected individuals, several developmental genes have been proposed as candidate genes for ONH [12,13].

We used custom-designed array comparative genomic hybridization (array-CGH) and whole genome sequencing (WGS) to perform a genome wide screen for large copy number variants (CNVs) as well as a targeted analysis of small CNVs, single nucleotide variants (SNVs), and indels in a population-based cohort of individuals with ONH in Stockholm, Sweden.

## Materials and methods

### Study population and participant demographics

In Stockholm, a population-based, cross-sectional cohort of children and young adults with ONH has been identified and phenotyped [1,5,6]. The diagnosis of ONH was confirmed when there was a small optic disc in combination with visual deprivation and/or a visual field defect [1]. In the present study cohort of 29 individuals, 18 individuals had bilateral ONH and 11 individuals had unilateral ONH. Fifteen were males and 14 females, and the median age at the time of the genetic analyses was 16.7 years (range 7.6–27 years). Six individuals (21%) were blind with a best-corrected decimal visual acuity (BCVA) of less than 0.05, classified according to the World Health Organization (WHO). Ten individuals (34%) had an intellectual disability and another four individuals had an autism spectrum disorder. Pituitary hormone deficiencies were diagnosed in nine individuals. Isolated ONH, without any combination with neurodevelopmental disorder, other congenital malformation, or pituitary hormone deficiency, was found in nine individuals (31%).

Genomic DNA derived from whole blood from the 29 individuals was collected according to standard protocols for genetic analyses. Written, informed consent for participation and publication of the results was obtained from the parents and from adolescents older than 15 years of age and the regional ethics committee in Stockholm (Regionala etikprövningsnämnden i Stockholm) granted approval of the study. The study was performed according to the Declaration of Helsinki.

### Array comparative genomic hybridization

A high-density custom array-CGH design was created using Agilents online web tool for array design, eArray. The used design was Agilent 2x400K HD-CGH microarray, consisting of approximately 400 000 oligonucleotide probes. Design and laboratory protocol for the array have previously been published [14]. Detection limits (log2 ratio) for duplications were set to 0.3 and for deletions 0.6. All aberrations called by Cytosure Interpret Software were manually inspected and classified according to the American College of Medical Genetics guidelines [15].

### Whole genome sequencing and variant validation

WGS was performed on 2.2 μg genomic DNA, extracted from whole blood according to standard protocols, at National Genomics Infrastructure (NGI) Stockholm using the Illumina XTen platform. The samples were prepared using a paired end (PE) PCR-free library prep, resulting in an average read depth of 30X and a mean insert size of 350 base pairs. Sequencing data was processed using the NGI-piper pipeline (https://github.com/johandahlberg/piper). Structural variants (balanced and unbalanced) were analysed using the FindSV pipeline (https://github.com/J35P312/FindSV), combining CNVnator [16] and TIDDIT [17]. Variant calling was performed using Bcftools [18] and freebayes [19], the resulting vcf files were merged using GATK combine variants and annotated using VEP [20]. Further, SNVs and indels were filtered with a maximum frequency of 0.01 in several normal variant databases including ExAC [21], the 1000 Genomes Project (1000G) [22], and the Swedish population frequency database (SweFreq) [23]. The variants were scored using SIFT (Sorting Intolerant From Tolerant) [24] and PolyPhen [25], the two most commonly used prediction tools for interpretation of genomic variation. Both algorithms predict whether a SNV has an effect on the protein structure based on sequence conservation and the physical properties of amino acids. The PolyPhen score ranges from 0.0 (tolerated) to 1.0 (deleterious), while the SIFT score ranges the opposite, from 0.0 (deleterious) to 1.0 (tolerated). The two models were interpreted together and deleterious predictions set to moderate or high were kept. Finally, the variants were manually filtered based on their quality through inspection using IGV [26] and previous literature. The remaining candidate variants were confirmed with Sanger sequencing and parental samples were analysed to determine the mode of inheritance. Only variants in genes previously implicated in human disease and with a CADD (combined annotation-dependent depletion) score [27] over 20 are presented and were classified according to the American College of Medical Genetics and Genomic guidelines [28].

The variants (CNVs and SNVs) were submitted to ClinVar database (https://www.ncbi.nlm.nih.gov/clinvar/) with accession numbers: SCV000681429, SCV000887490, SCV000681422, SCV000681432, SCV000681428, SCV000681434, SCV000681433, SCV000681423, SCV000681425, SCV000681430, SCV000681431, SCV000681427, and were named according to the following NCBI GenBank reference transcript accession numbers: *SOX5*, NM_152989.4; *KIF7*, NM_198525.2; *COL4A1*, NM_001845.5; *COL4A2*, NM_001846.3; *OPA1*, NM_015560.2; *SPG7*, NM_003119.3; *CYP26A1*, NM_057157.2; *CYP26C1*, NM_183374.2; *UBE3B*, NM_130466.3.

### Statistical analysis

Descriptive statistics were performed using STATISTICA 13 (StatSoft Inc, Tulsa, Oklahoma, USA).

## Results

### Identification of one rare CNV in *SOX5* in one case with ONH

**Fig 1:**
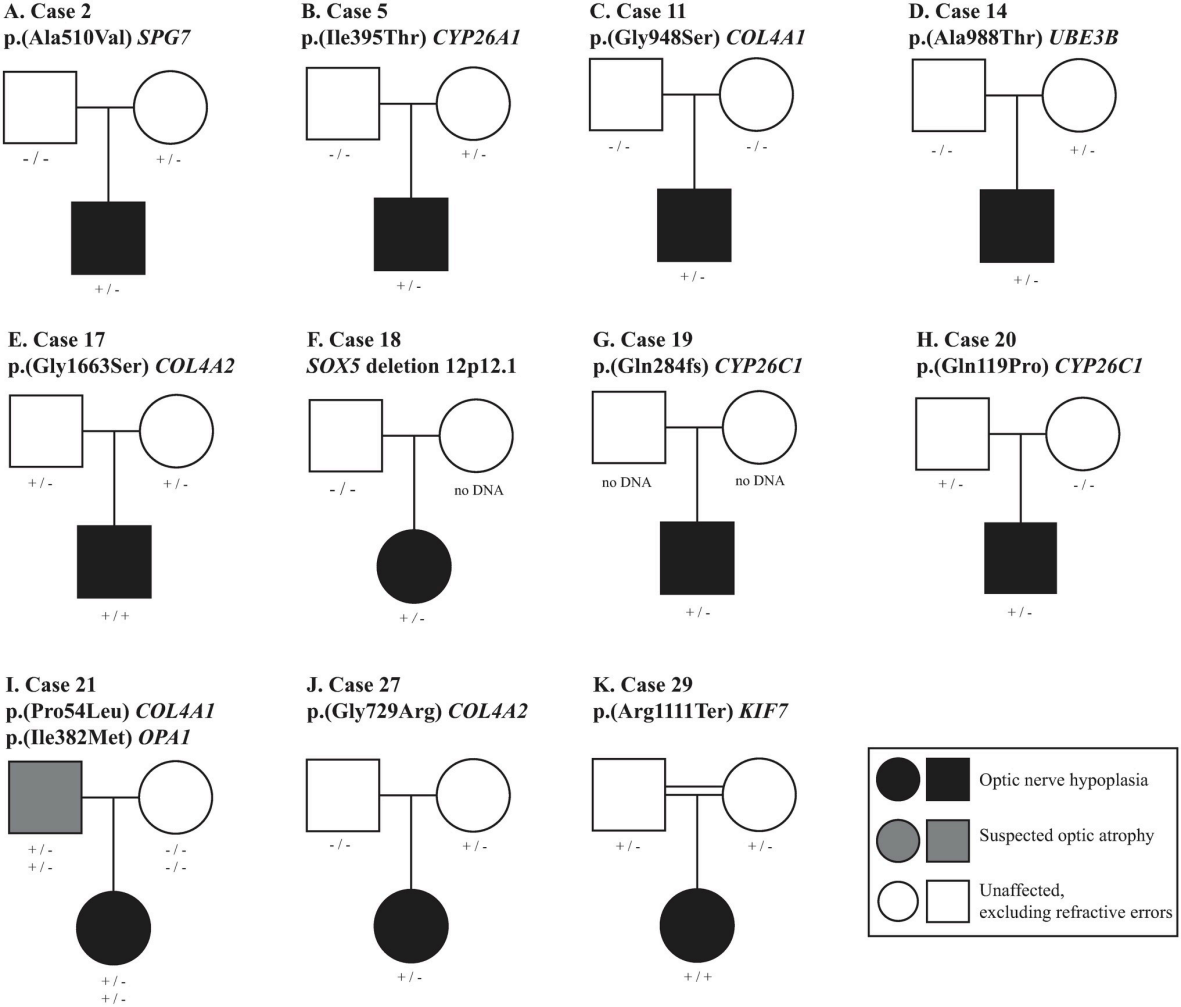
Segregation analyses in 10 of 11 families with rare genomic variants. DNA samples from 19 parents were available for segregation analysis and revealed that eight SNVs in eight individuals were inherited. The SNV in Case 11 was *de novo*. The CNV identified in Case 18 had only one parental sample available and the inheritance could not be established. A diagnosis of optic nerve hypoplasia is indicated in black, suspected optic atrophy is indicated in grey, and no ophthalmological disorder, excluding refractive errors, is indicated in white.

**Fig 2:**
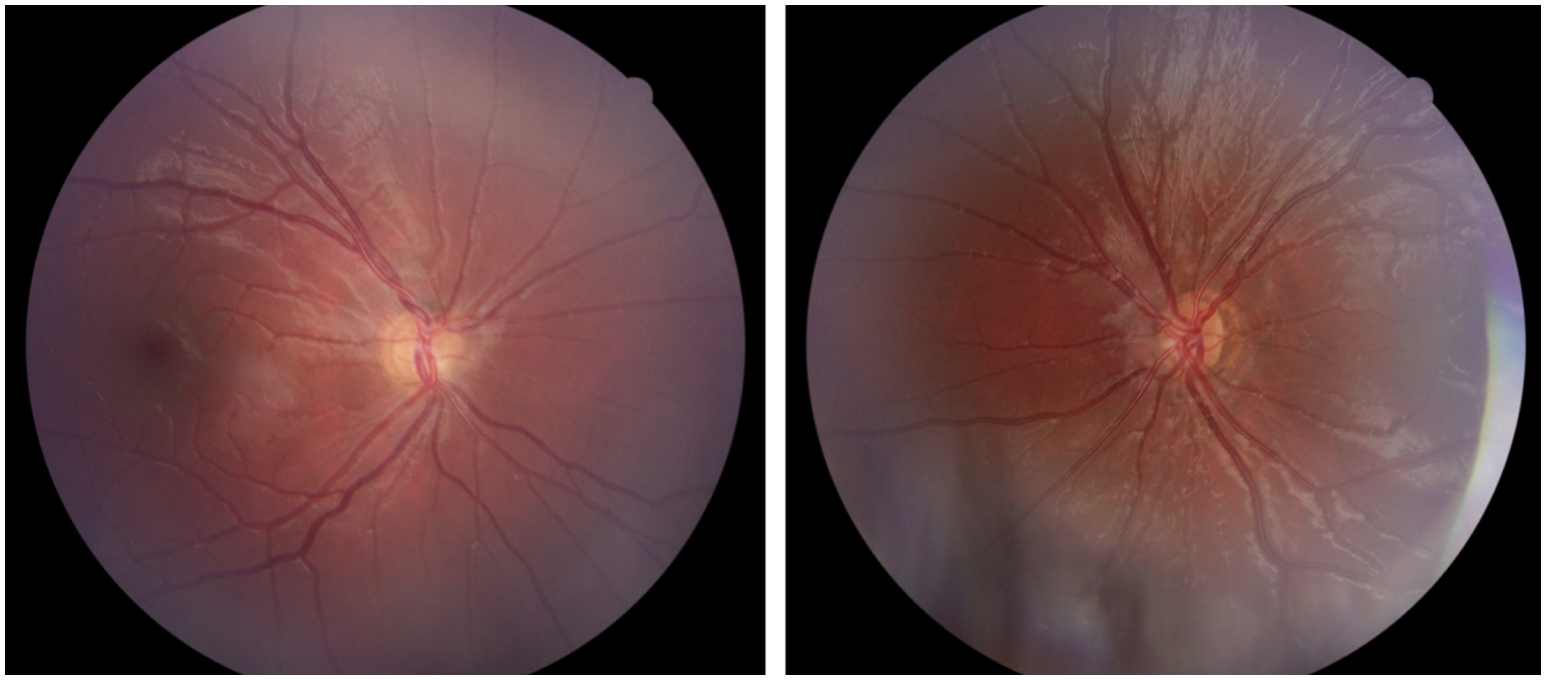
Fundus photographs from Case 18 with bilateral optic nerve hypoplasia. Case 18 was born full term and developmental delay was noticed at one year of age. She was diagnosed with moderate intellectual disability and has autistic traits. She had strabismus and bilateral optic nerve hypoplasia was diagnosed at three years of age. The BCVA was 0.12 right and left eye at six years of age.

In the cohort of 29 individuals with ONH, array-CGH analysis identified one rare CNV (Case 18, Table 1, Fig 1). The CNV was a heterozygous deletion of 341 kb at 12p12.1 (chr12:23432684–23773692, GRCh37/Hg19) involving exons 7–18 of *SOX5* (MIM 604975). *SOX5* encodes a transcription factor that is important for neurogenesis [29]. Interestingly, both mutations and similar deletions of *SOX5* have previously been described in Lamb-Schaffer syndrome (MIM 616803), an autosomal dominant syndrome with varying phenotypes including ONH, optic atrophy (MIM 165500), developmental delay, behavioural problems, poor expressive speech, mild dysmorphic features, and skeletal abnormalities [30,31]. The affected individual (Case 18) has bilateral ONH, moderate visual impairment, intellectual disability, autistic traits, and a small ventricular septal defect suggestive of Lamb-Schaffer syndrome (Fig 2). DNA was only available from the father and he did not carry the deletion. Subsequently, the *SOX5* deletion was assessed as pathogenic.

**Table 1:** Clinical phenotypes and identified candidate variants in 11 optic nerve hypoplasia cases.

Case	Sex	Age* (y)	Gene	Inheritance	ONH	Neuro	Hormone deficiency	Other malformations
**2**	M	24	*SPG7*	Maternal	Bilat Blind	ID,CP,EP	-	Microcephaly, WMA, facial asymmetry
**5**	M	13	*CYP26A1*	Maternal	Bilat	-	MPHD	Pituitary abnormality
**11**	M	10	*COL4A1*	De novo	Bilat Blind	ID,CP,EP	-	CC hypoplasia, CD, calcification, WMA, microcephaly
**14**	M	14	*UBE3B*	Maternal	Bilat	-	-	Unilat syndactyly toes
**17**	M	18	*COL4A2*	Maternal, paternal	Bilat	ASD, hyposmia	MPHD	Pituitary and jaw abnormalities
**18**	F	11	Het del *SOX5* exon 7–18	Not paternal	Bilat	ID, autistic traits	-	VSD
**19**	M	18	*CYP26C1*	Ni	Unilat	-	-	-
**20**	M	11	*CYP26C1*	Paternal	Unilat	-	-	-
**21**	F	14	*COL4A1 OPA1*	Paternal, paternal	Unilat	-	Precocious puberty	-
**27**	F	16	*COL4A2*	Maternal	Unilat	ID	-	Hypertelorism
**29**	F	23	*KIF7*	Maternal, paternal	Bilat	ID	-	CC agenesis, SP agenesis, MTS, colpocephaly, arachnoid cyst, macrocephaly, hypertelorism, ovarian cysts, liver and uterus abnormalities, bowel malrotation, unilat syndactyly toes

*Age at the time of the genetic analyses. ONH, optic nerve hypoplasia; Neuro, neurological dysfunction; Bilat, bilateral; ID, intellectual disability; CP, cerebral palsy; EP, epilepsy; WMA, white matter abnormalities; MPHD, multiple pituitary hormone deficiency; CC, corpus callosum; CD, cortical destruction; Unilat, unilateral; ASD, autism spectrum disorder; Het del, heterozygous deletion; VSD, ventricular septal defect; Ni, no information; SP, septum pellucidum; MTS, molar tooth sign.

### Whole genome sequencing identifies 11 rare SNVs in 10 ONH cases

#### SNVs in *COL4A1* and *COL4A2*

**Fig 3:**
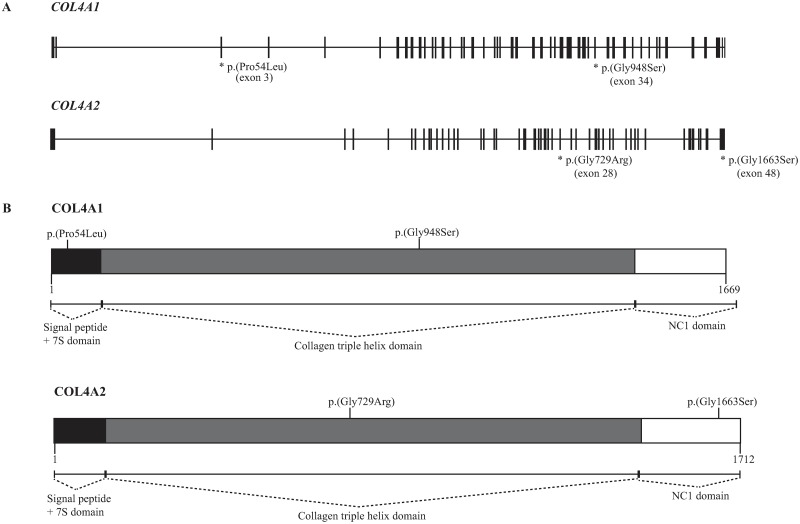
Rare variants in *COL4A1* and *COL4A2*. Rare variants in *COL4A1* and *COL4A2* were identified in 4/29 (14%) individuals with optic nerve hypoplasia (A). Of these four variants, two affected the collagen triple helix domain of COL4A1 or COL4A2, one affected the 7S domain of COL4A1, and one affected the noncollagenous (NC1) domain of COL4A2 (B).

WGS of the 29 individuals with ONH resulted in 11 rare SNVs identified in 10 ONH cases (Tables 1 and 2, Fig 1). Of these, two variants were located in *COL4A1* (MIM 120130) and two variants in *COL4A2* (MIM 120090) (Fig 3).

**Table 2:** Rare single nucleotide variants identified in the optic nerve hypoplasia cohort.

Case	Gene	cDNA change	Amino acid change	rsID	Zygosity	CADD [27]	ExAC [21]	SweFreq [23]
**2**	*SPG7*	c.1529C>T	p.(Ala510Val)	rs61755320	Het	32	0.0036	0.0035
**5**	*CYP26A1*	c.1184T>C	p.(Ile395Thr)	rs757601865	Het	23.8	0	0
**11**	*COL4A1*	c.2842G>A	p.(Gly948Ser)	-	Het	25.6	0	0
**14**	*UBE3B*	c.2962G>A	p.(Ala988Thr)	rs753221661	Het	34	0.0001	0
**17**	*COL4A2*	c.4987G>A	p.(Gly1663Ser)	rs12877501	Hom	23.7	0.0009	0.001
**19**	*CYP26C1*	c.845_851dup CCATGCA	p.(Gln284fs)	rs565866662	Het	34	0.0029	0.007
**20**	*CYP26C1*	c.356A>C	p.(Gln119Pro)	rs201284617	Het	23.5	0.0001	0.0055
**21**	*COL4A1*	c.161C>T	p.(Pro54Leu)	rs34004222	Het	26.5	0.0035	0.0035
**21**	*OPA1*	c.1146A>G	p.(Ile382Met)	rs143319805	Het	24.4	0.0008	0
**27**	*COL4A2*	c.2185G>A	p.(Gly729Arg)	rs201058867	Het	24.2	0.0004	0.0015
**29**	*KIF7*	c.3331C>T	p.(Arg1111Ter)	rs778139192	Hom	43	0	0

CADD, combined annotation-dependent depletion; Het, heterozygous; Hom, homozygous.

Three of the variants in *COL4A1* or *COL4A2* were very interesting. In Case 11, we identified a rare variant *COL4A1* p.(Gly948Ser) confirmed to be *de novo* by segregation studies. The same variant has previously been reported and described as pathogenic in an individual with porencephaly, calcification, cerebral palsy, epilepsy, and intellectual disability [32]. Case 11 had similar symptoms except porencephaly (Table 1). He was born full term and was diagnosed with bilateral ONH at four months of age due to lack of fixation. Besides blindness, he had infantile spasms and continued to have epilepsy, but also severe bilateral spastic cerebral palsy as well as severe intellectual disability. This finding is further evidence of the pathogenicity of the *COL4A1* p.(Gly948Ser) mutation.

**Fig 4:**
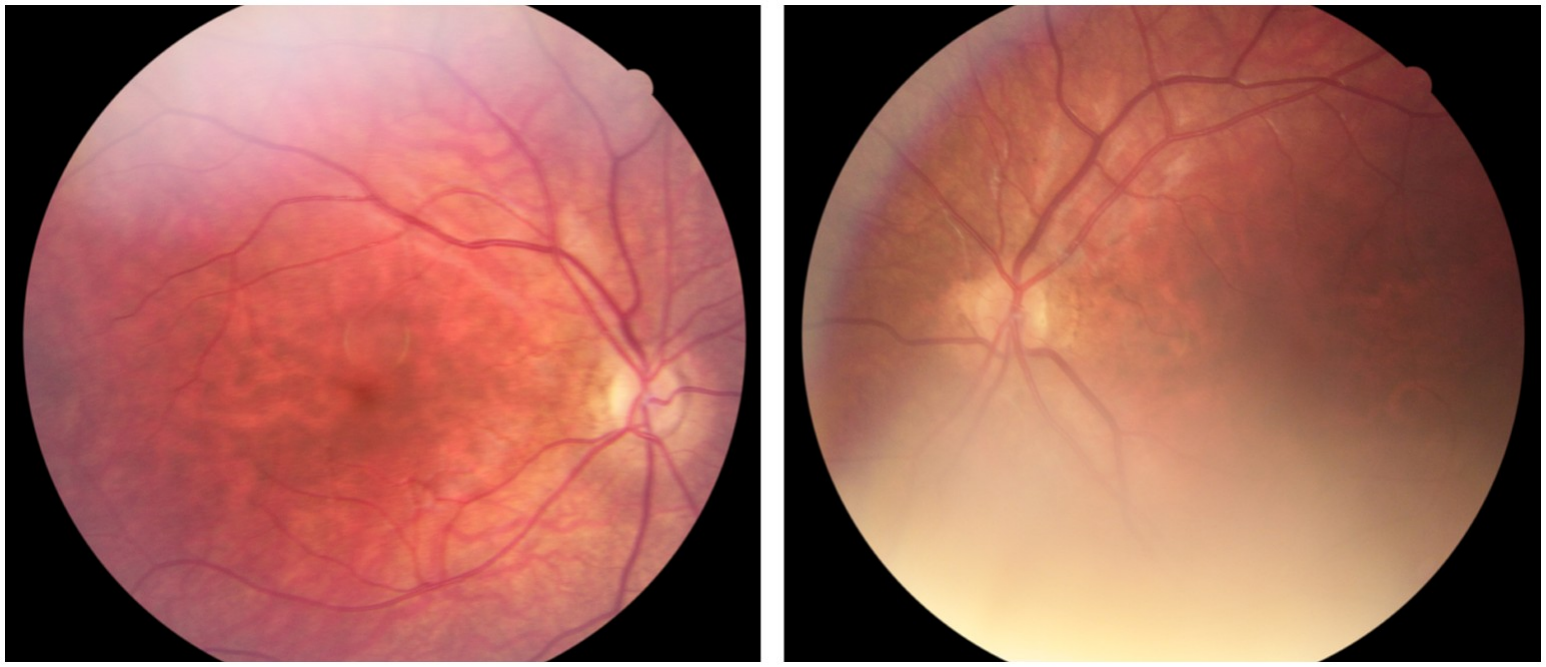
Fundus photographs from Case 17 with bilateral optic nerve hypoplasia. Case 17 was born full term and due to nystagmus bilateral optic nerve hypoplasia was diagnosed at one month of age. He was visually impaired with a BCVA of 0.15 right and 0.03 left eye at 12 years of age. Extreme thirst and weight gain at three months of age revealed a diabetes insipidus. In addition, he had adrenocorticotropic hormone deficiency. At ten years of age he was diagnosed with an autism spectrum disorder, but he has normal intelligence. Furthermore, he has hyposmia, mild gross motor function impairment, and jaw abnormalities.

**Fig 5:**
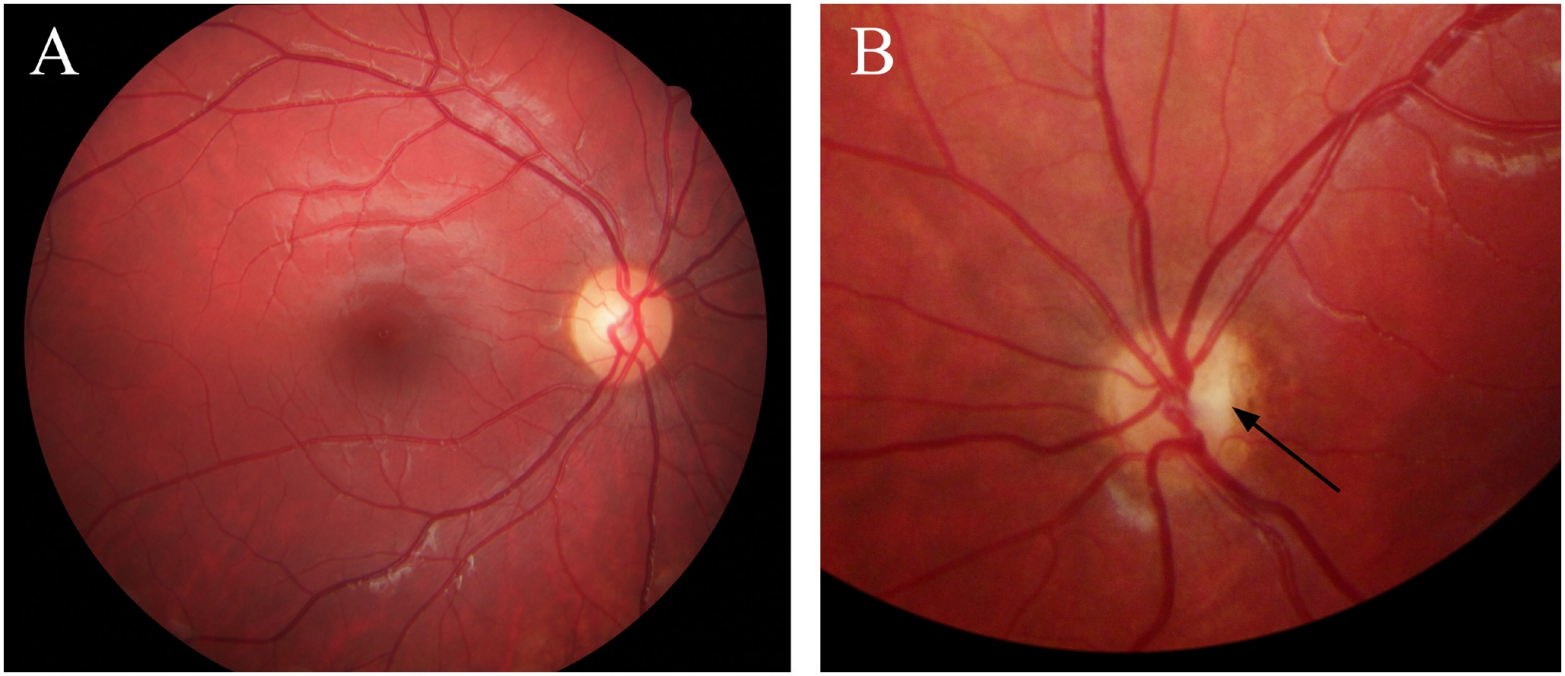
Fundus photographs from Case 21 with unilateral optic nerve hypoplasia left eye. Fundus photographs from Case 21 with unilateral optic nerve hypoplasia and heterozygous missense variants in *COL4A1* and *OPA1* showing (A) a normal optic disc in the right eye and (B, magnified image) optic nerve hypoplasia in the left eye with a small optic disc (arrow) and a surrounding peripapillary atrophy. Case 21 was born full term and was treated with patching from 11 months of age because of strabismus (esotropia left eye). She was lost to follow-up and unilateral optic nerve hypoplasia was diagnosed after the visual screening at four years of age. The BCVA was 1.0 right and 0.03 left eye at 15 years of age. She had a precocious puberty from seven years of age and is normally developed.

**Fig 6:**
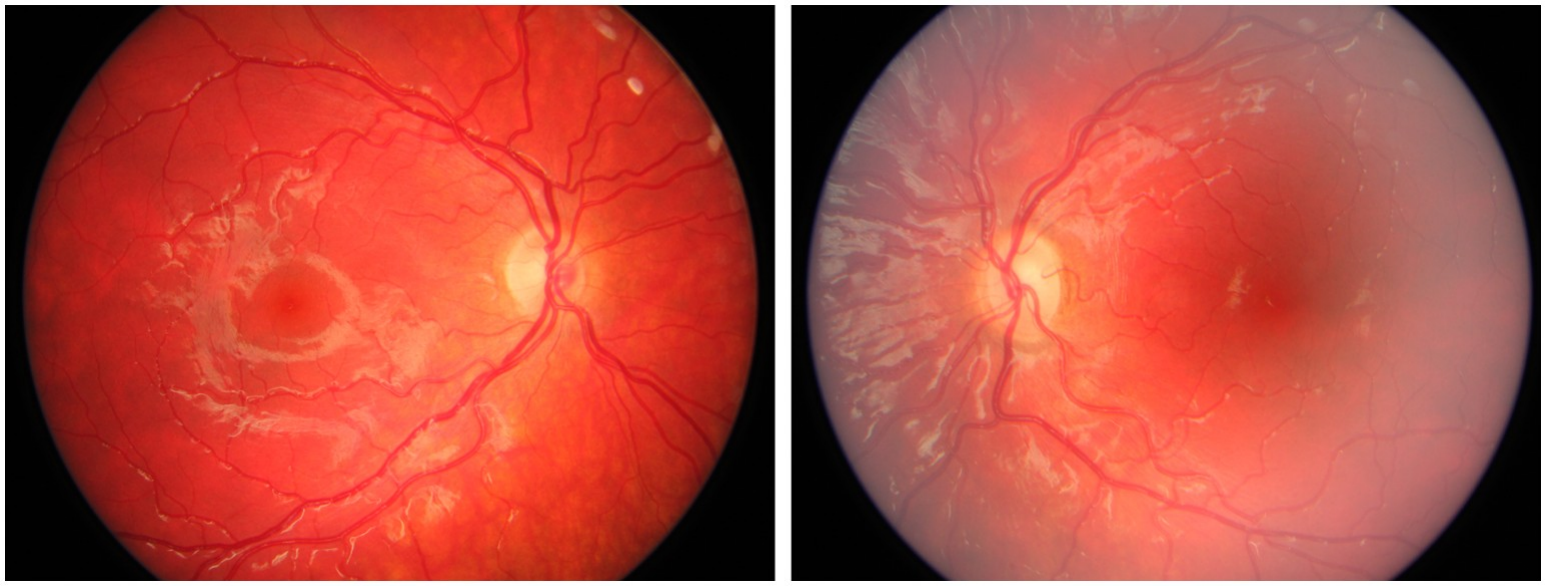
Fundus photographs from Case 27 with unilateral optic nerve hypoplasia right eye. Case 27 was born at 37 weeks of gestation. She developed strabismus (exotropia right eye) and unilateral optic nerve hypoplasia was diagnosed at two years of age. The BCVA was 0.5 right and 1.0 left eye at 11 years of age. She has a mild intellectual disability.

The second variant of interest was found in Case 17 who had a homozygous variant in *COL4A2* p.(Gly1663Ser) with each allele inherited from two unrelated parents (Figs 1 and 4). Interestingly, there are no homozygous individuals with this variant reported in the ExAC database [21]. The third individual (Case 21) had two rare variants, a heterozygous missense variant in *COL4A1* p.(Pro54Leu) and a heterozygous missense variant in *OPA1* p.(Ile382Met) (MIM 605290) (Fig 5). Both variants were inherited from the father, who had a suspected traumatic unilateral optic atrophy. Moreover, the fourth individual (Case 27) had a heterozygous missense variant in *COL4A2* p.(Gly729Arg) inherited from a healthy mother (Fig 6).

In total, 4/11 (36%) of all the rare SNVs identified were in *COL4A1* or *COL4A2*, and 4/29 (14%) of the individuals in the cohort carried a rare missense variant in either of these two genes. Constraint metrics retrieved from the ExAC browser indicated a z-score for missense variants of 2.46 in *COL4A1* and 1.76 in *COL4A2*. Hence, the increased number of missense variants in these particular genes could not directly be explained by decreased constraint.

#### Pathogenic homozygous SNV in *KIF7*

**Fig 7:**
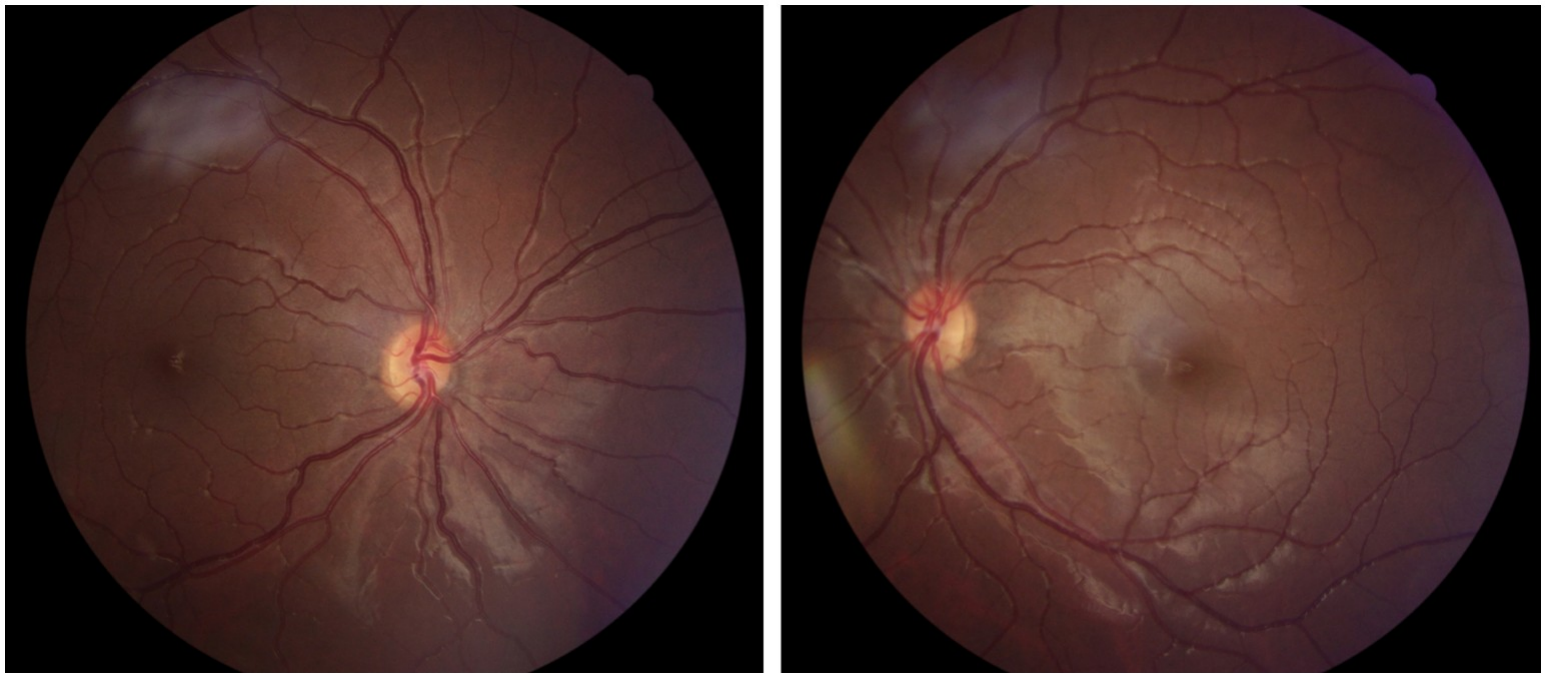
Fundus photographs from Case 29 with bilateral optic nerve hypoplasia. Case 29 was born full term and came to Sweden from Iraq at four years of age. She had strabismus (exophoria left eye) and multiple brain malformations, including thin optic nerves, on magnetic resonance imaging. However, bilateral optic nerve hypoplasia was not diagnosed until she was 17 years of age. At 18 years of age the BCVA was 0.8 right and 0.5 left eye. She was further diagnosed with a moderate intellectual disability and has mild motor impairments.

Another striking finding was a homozygous nonsense variant in *KIF7* p.(Arg1111Ter) (MIM 611254) in Case 29, inherited from both of her consanguineous parents. The *KIF7* variant has previously been reported as pathogenic in an individual with Joubert syndrome (MIM 213300) [33]. The phenotype in Case 29 is concordant with Joubert syndrome with molar tooth sign on magnetic resonance imaging, ataxia, impairments in gross and fine motor function, and intellectual disability. In addition, Case 29 has craniofacial features fitting acrocallosal syndrome (MIM 200990) [34], namely macrocephaly, prominent forehead, hypertelorism, downslanting palpebral fissures as well as wide and depressed nasal bridge (Table 1, Fig 7). Acrocallosal syndrome is an autosomal recessive neurodevelopmental ciliopathy often caused by *KIF7* mutations and phenotypically overlaps with Joubert syndrome [34].

In summary, three of the SNVs (*COL4A1* p.(Gly948Ser), *COL4A2* p.(Gly1663Ser), and *KIF7* p.(Arg1111Ter)) were assessed as pathogenic or likely pathogenic, and the remaining eight SNVs were heterozygous variants of unknown clinical significance.

## Discussion

In the present study, we performed a systematic screening for rare genomic variants in 29 individuals with ONH and identified 11 rare SNVs in ten individuals and one rare CNV in one individual. This is the first WGS study of a cohort with ONH and the results show that there is a genetic heterogeneity in ONH and indicates that genetic causes of ONH are not rare. The diagnostic yield of pathogenic or likely pathogenic variants in individuals with ONH using WGS was 4/29 (14%), all of whom had non-isolated ONH. Consequently, in the group of non-isolated ONH, 4/20 (20%) had a presumed genetic cause.

SNVs in *COL4A1* and *COL4A2* genes were frequent in our cohort (4/29, 14%). In particular, two of the rare variants were especially interesting. They were the heterozygous *de novo* variant *COL4A1* p.(Gly948Ser) previously described as pathogenic and the homozygous variant *COL4A2* p.(Gly1663Ser) [32]. *COL4A1* and *COL4A2* encode, respectively, the alpha-1 and alpha-2 subunits of collagen type IV. Together they form a heterotrimer of two alpha-1 chains and one alpha-2 chain. Collagen type IV is essential for basement membranes and is involved in cell adhesion, migration, proliferation, and differentiation [35]. The two genes are arranged head-to-head on opposite strands at 13q34 with a common bidirectional promotor [35]. Mutations in *COL4A1* have been associated with small-vessel brain disease including porencephaly, schizencephaly, cerebrovascular disease, ophthalmological disorders (retinal arterial tortuosity, congenital cataract, anterior segment dysgenesis, glaucoma, microphthalmia/anophthalmia, optic atrophy, and ONH), but also with renal and muscular abnormalities [32,36,37]. There is an autosomal dominant pattern of inheritance, broad phenotypic variation with reduced penetrance, and a wide heterogeneity of symptoms within families. Similarly, mutations in *COL4A2* have been associated with porencephaly, intracerebral haemorrhages, optic atrophy, and ONH [37–41]. Animal studies further support their involvement in ONH and the variability of phenotypic penetrance [42]. *Col4a1*^*+/Δex41*^ mutant mice develop ONH, but this is dependent on the genetic background of these mice [43]. In addition, these mice show changes in the retinal inner limiting membrane, suggesting that abnormalities of the basement membranes may cause ONH [43]. However, despite the evidence for a role of *Col4* paralogues in ONH, only a few ONH cases with mutations in *COL4A1* and *COL4A2* are described in the literature [32,38].

Here we report two rare variants in *COL4A1* and two rare variants in *COL4A2*. The location of the variants in *COL4A1* and *COL4A2* seems to be of major importance. Two of these variants were located in the triple helix domain, one in the amino-terminal 7S domain of COL4A1, and one in the carboxy-terminal noncollagenous (NC1) domain of COL4A2 (Fig 3). The two variants located in the triple helix domain resulted in the substitution of a glycine (Fig 3). The first was the heterozygous *de novo* variant *COL4A1* p.(Gly948Ser) and the second was the heterozygous variant *COL4A2* p.(Gly729Arg). In the triple helix domain, the triple amino acid repeats with glycine in every third position are essential for the proper triple helical formation and mutations in this region are known to disrupt the normal folding and protein function [44]. Hence, substitutions of the glycine in these repeats have been predicted to be pathogenic and are assumed to have a dominant-negative effect on the protein [35,37]. In contrast, the homozygous variant *COL4A2* p.(Gly1663Ser) were located in in the NC1 domain, which has a prominent role in regulating the stoichiometry of the α1α1α2 heterotrimer [35]. One may hypothesise that this homozygous variant could affect the composition of the heterotrimer and subsequently, the function of collage typ IV.

Previous research has shown a high *de novo* mutation rate in *COL4A1* and *COL4A2* as well as inherited pathogenic or likely pathogenic mutations [37]. In our cohort, only one *COL4A1* variant was shown to be *de novo*. Moreover, because of the broad phenotypic variation with reduced penetrance, the inherited heterozygous rare variants *COL4A1* p.(Pro54Leu) and *COL4A2* p.(Gly729Arg) could not be assessed as likely benign and were assessed as variants of unknown clinical significance. The substitution of a glycine in the triple helix domain in the variant *COL4A2* p.(Gly729Arg) even suggests that it may be pathogenic. Consequently, functional studies would be helpful to understand the clinical significance of the inherited variants identified in this study.

In Case 21, in addition to the *COL4A1* variant, an *OPA1* variant p.(Ile382Met) was identified. Mutations in *OPA1* are usually associated with optic atrophy 1, but it has previously been suggested that *OPA1* may also be involved in ONH [45]. The variant identified in Case 21 is enriched in individuals with autosomal dominant optic atrophy, but is also present in both the dbSNP and ExAC databases (rs143319805) [46]. In two other optic atrophy studies, the *OPA1* variant p.(Ile382Met) was identified in a compound heterozygous state in a total of five cases, and heterozygous carriers were an asymptomatic father and a mother with myopia and mild sensorineural hearing loss [47,48]. The authors hypothesised that this variant may have a semi-dominant mode of inheritance [48]. Case 21 has a clinically significant unilateral ONH shown in Fig 5. The rare variants in *OPA1* and *COL4A1* were inherited from the father who had strabismus as a child, suffered from a traumatic macular haemorrhage in the left eye as an adolescent and went through cataract surgery in adulthood (Fig 1). He was never diagnosed with ONH, but suspected to have a traumatic optic atrophy in his left eye.

In Case 29, we found a pathogenic homozygous nonsense variant in *KIF7* p.(Arg1111Ter). Mutations in *KIF7* are associated with the ciliopathy Joubert syndrome [33] and especially acrocallosal syndrome [34], which both fit with the patient’s symptoms. The kinesin KIF7 is an important regulator for the sonic hedgehog signaling pathway at the primary cilium [49]. Sonic hedgehog signaling is essential for patterning and brain development [49]. Furthermore, prenatal ethanol exposure causes ONH by inhibition of sonic hedgehog signaling in retinal progenitor cells in mice, resulting in failure to extend axons to the optic nerve [50]. Accordingly, both genetic and environmental factors may affect the sonic hedgehog signaling pathway and cause ONH. However, in Case 29, ONH could be a result of a primary optic nerve malformation and/or secondary to trans-synaptic degeneration due to the other brain malformations.

Moreover, rare SNVs were identified in four additional genes in five individuals; *SPG7* (1/29), *CYP26A1* (1/29), *CYP26C1* (2/29), *UBE3B* (1/29), and one rare CNV affecting *SOX5* (1/29) was detected. The variant identified in *SPG7* p.(Ala510Val) (MIM 602783) has previously been described in individuals with spastic paraplegia 7 (MIM 607259), and another *SPG7* variant, c.1232A>C, p.(Asp411Ala), segregated with the disease in a family with an autosomal dominant optic neuropathy [51]. The individual carrying the heterozygous *SPG7* variant in this study (Case 2) was born full term and developed strabismus and nystagmus, but was not diagnosed with bilateral ONH until he was 17 years of age despite blindness. In addition to ONH, he has severe intellectual disability, bilateral spastic cerebral palsy, epilepsy, microcephaly, and facial asymmetry. The specific p.(Ala510Val) variant is commonly described in autosomal recessive spastic paraplegia, nevertheless, in a study of 285 individuals with spastic paraplegia, a dominant effect was suggested for the p.(Ala510Val) [52]. In Case 2, the variant *SPG7* p.(Ala510Val) was inherited from a healthy mother. Nonetheless, considering previous evidence of involvement in brain dysfunction, we speculate that this variant could be a partial explanation of the patient’s symptoms, including ONH, and it was assessed as a variant of unknown clinical significance.

**Fig 8:**
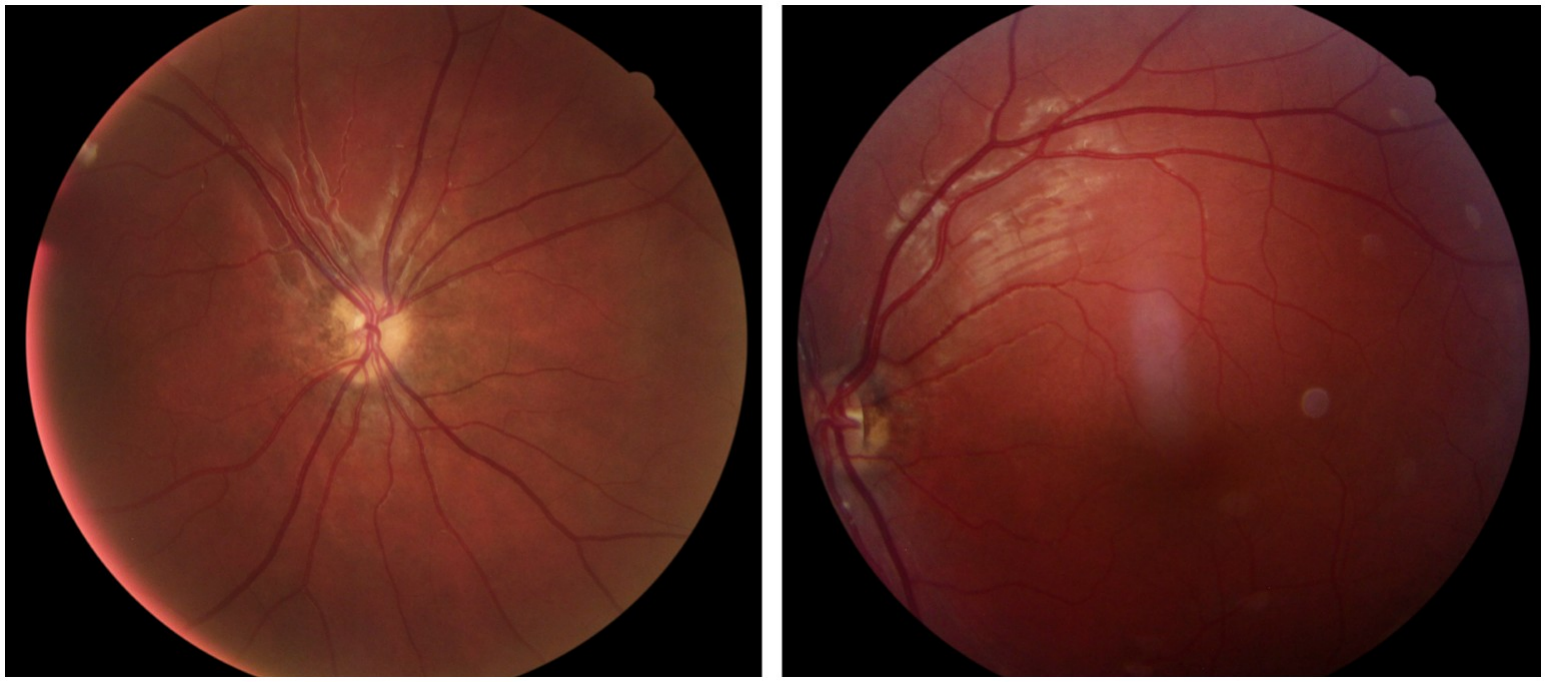
Fundus photographs from Case 5 with bilateral optic nerve hypoplasia. Case 5 was born full term and had neonatal hypoglycemia and seizures. Bilateral optic nerve hypoplasia was diagnosed at two months of age due to lack of fixation and nystagmus. At the same age he was diagnosed with adrenocorticotropic hormone deficiency, and later on he was also treated for growth hormone deficiency and central hypothyroidism. The BCVA was 0.125 right and left eye at seven years of age. He is normally developed.

**Fig 9:**
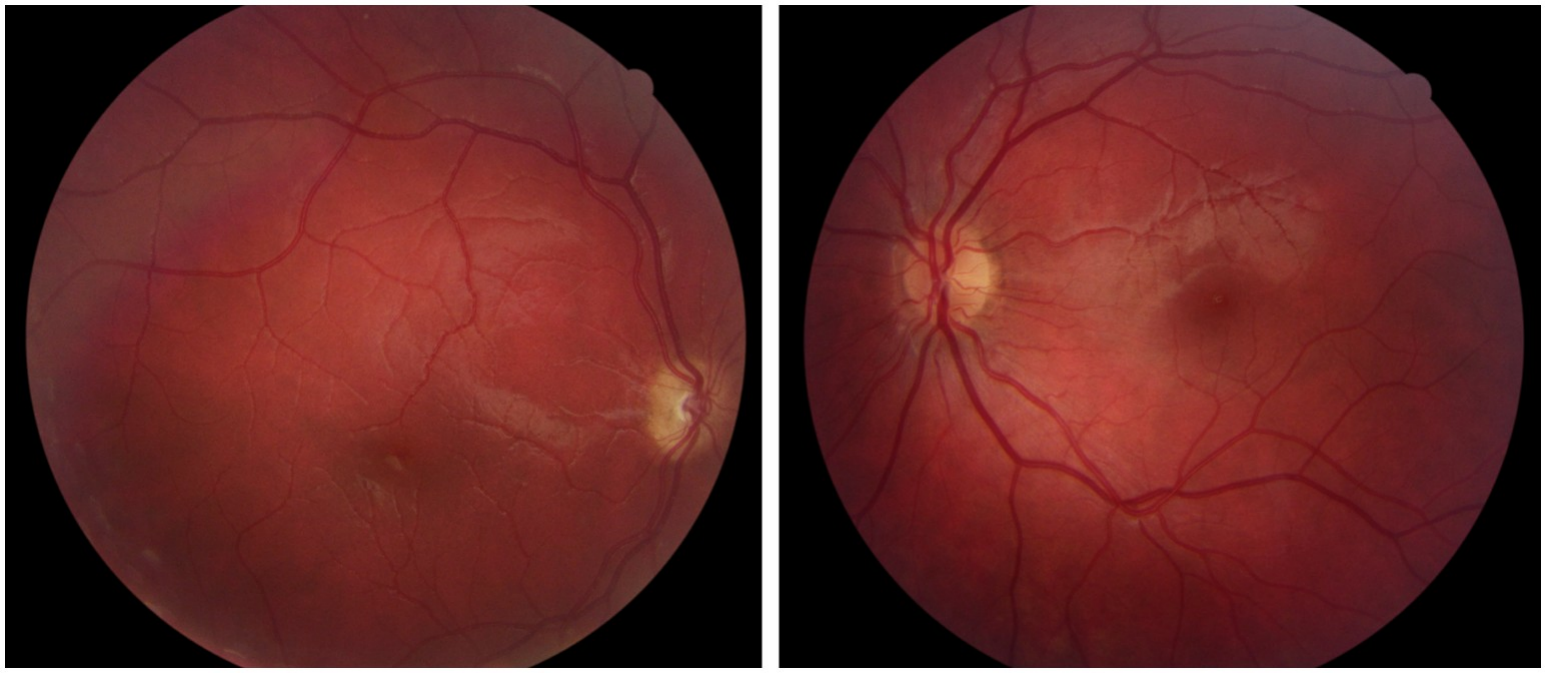
Fundus photographs from Case 19 with unilateral optic nerve hypoplasia right eye. Case 19 was born full term. He had severe strabismus (esotropia right eye) and was diagnosed with unilateral optic nerve hypoplasia at two months of age. He is blind in his right eye, but the BCVA was 1.0 left eye at 12 years of age. He is normally developed.

**Fig 10:**
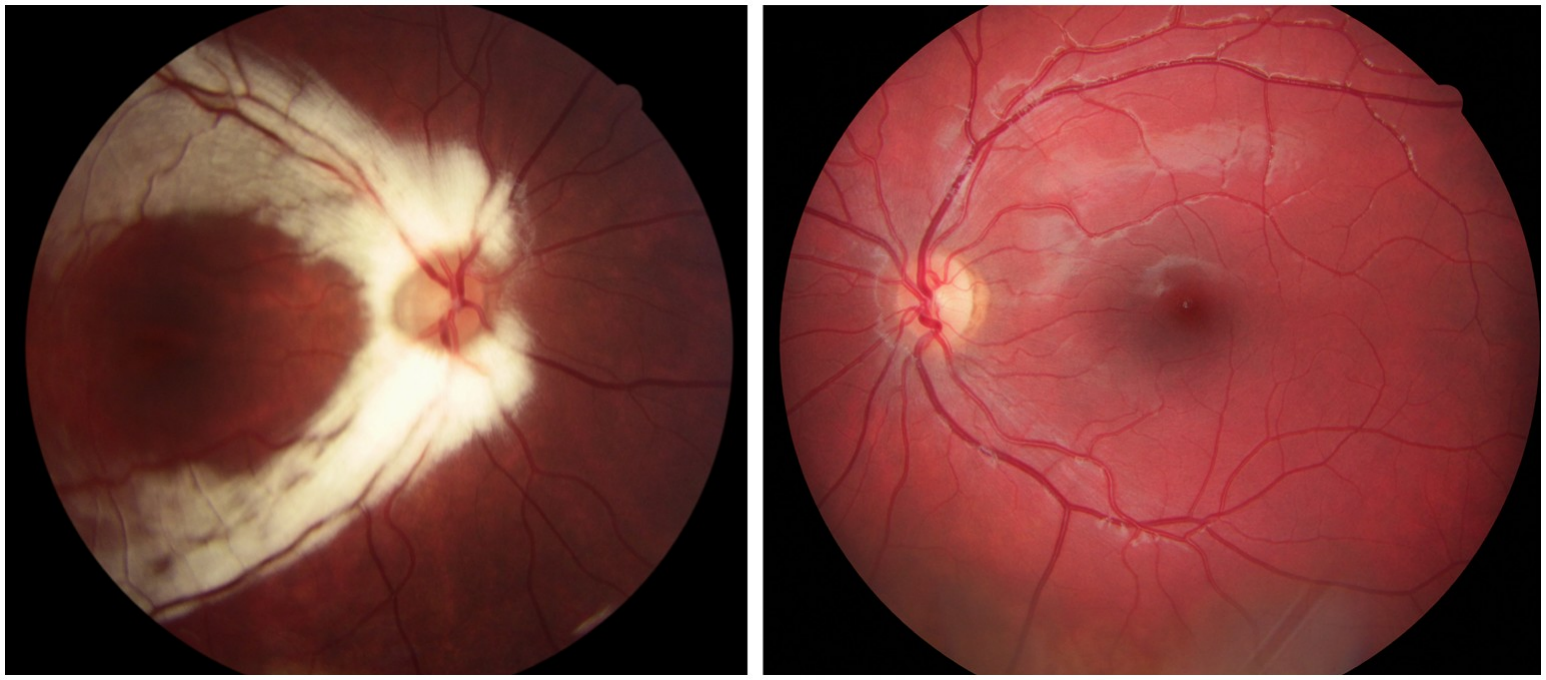
Fundus photographs from Case 20 with unilateral optic nerve hypoplasia right eye. Case 20 was born full term and unilateral optic nerve hypoplasia was discovered at the ophthalmology department after detection at the visual screening at four years of age. He had strabismus (esotropia right eye) and the BCVA was 0.1 right and 1.0 left eye at five years of age. He has normal intelligence.

**Fig 11:**
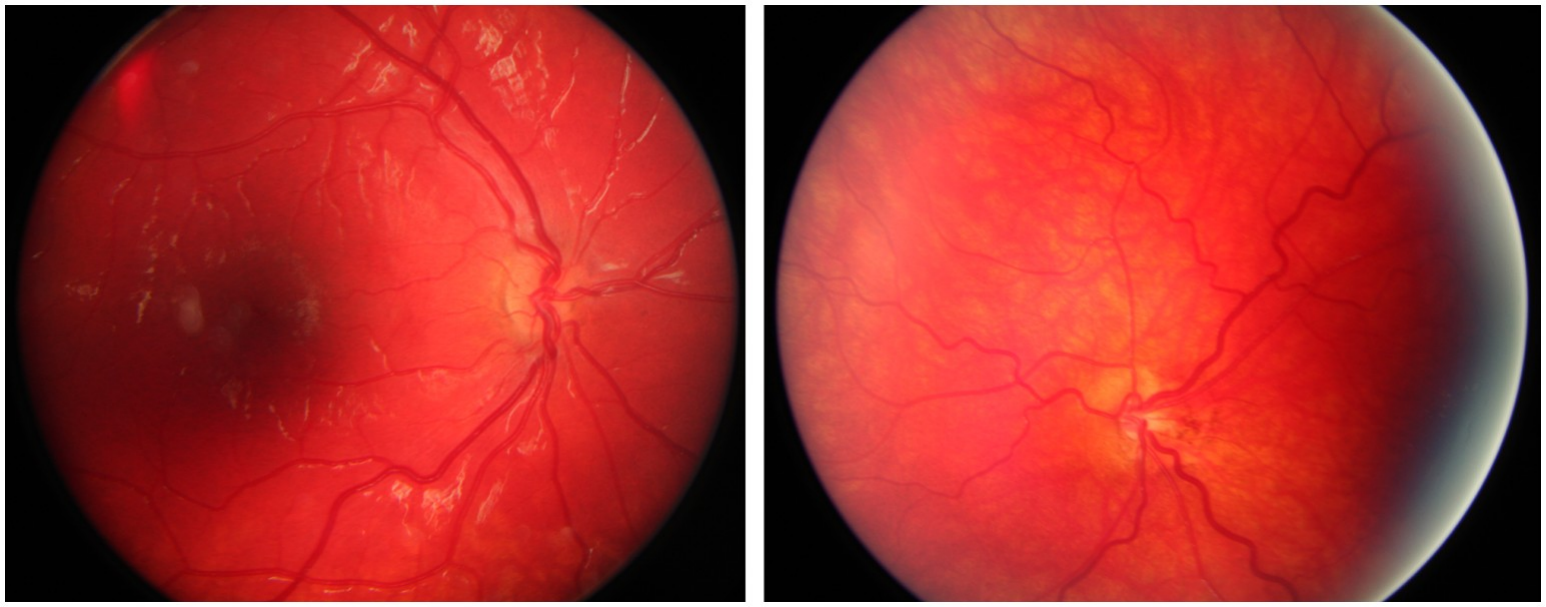
Fundus photographs from Case 14 with bilateral optic nerve hypoplasia. Case 14 was born preterm at 27 weeks of gestation. He had strabismus and was diagnosed with left sided optic nerve hypoplasia at 13 months of age, which on closer assessment proved to be bilateral optic nerve hypoplasia when he was 13 years of age. The BCVA was 1.0 right and 0.1 left eye at eight years of age, but he had a significant visual field defect in his right eye. He is normally developed.

Finally, deletions of *CYP26A1* (MIM 602239) and *CYP26C1* (MIM 608428) have previously been implicated in both nonsyndromic bilateral and unilateral optic nerve aplasia [53], and mutations in *UBE3B* (MIM 608047) have been described in Kaufman Oculocerebrofacial Syndrome (MIM 244450) [54]. However, since the three variants where parental DNA was available showed inheritance from a healthy parent, they were assessed as less likely to be the cause of the ONH phenotype (Figs 1 and 8–11).

Likely, ONH can be caused of monogenic, polygenic and environmental factors, single or in combinations. Lubinsky previously hypothesised that septo-optic dysplasia (MIM 182230), including ONH, is a result of a vascular disruptive sequence, possibly involving the proximal trunk of the anterior cerebral artery [55]. Risk factors such as early gestational vaginal bleeding and use of vasoconstrictors (tobacco and cocaine) may increase the risk of a vascular insult [56], and so could mutations in *COL4A1* or *COL4A2*. We speculate that the broad phenotype seen in children with ONH may be explained by the disease gene and the overall mutational burden, modified by the regulatory landscape and environmental factors. This could also explain why so few family members are affected and the reduced penetrance seen in many of the candidate genes. We used WGS to analyse protein-coding sequences of known disease causing genes, but it would also be very interesting to look at novel genes and non-coding sequences, including regulatory elements. It is reasonable to assume that genetic mutations in these parts may contribute to the broad phenotype and reduced penetrance, and this should be further studied. Chen *et al* have published an excellent review of genetic causes of ONH which highlighted 16 genes [12]. To this list we suggest to add *COL4A1* and *COL4A2*, which seem to be important genetic contributors to ONH.

In conclusion, the present study elucidates the genetic heterogeneity in ONH and indicates that genetic causes of ONH are not rare. We conclude that genetic testing is valuable in a substantial proportion of the individuals with ONH, especially in cases with non-isolated ONH. With the increased use of WGS more individuals with ONH should be able to receive a genetic diagnosis in the future.
